# Involvement of the 14-3-3 Gene Family in Autism Spectrum Disorder and Schizophrenia: Genetics, Transcriptomics and Functional Analyses

**DOI:** 10.3390/jcm9061851

**Published:** 2020-06-13

**Authors:** Bàrbara Torrico, Ester Antón-Galindo, Noèlia Fernàndez-Castillo, Eva Rojo-Francàs, Sadaf Ghorbani, Laura Pineda-Cirera, Amaia Hervás, Isabel Rueda, Estefanía Moreno, Janice M. Fullerton, Vicent Casadó, Jan K. Buitelaar, Nanda Rommelse, Barbara Franke, Andreas Reif, Andreas G. Chiocchetti, Christine Freitag, Rune Kleppe, Jan Haavik, Claudio Toma, Bru Cormand

**Affiliations:** 1 Departament de Genètica, Microbiologia i Estadística, Facultat de Biologia, Universitat de Barcelona, Prevosti Building, floor 2, Av. Diagonal 643, 08028 Barcelona, Spain; barticoa@gmail.com (B.T.); eantongalindo@ub.edu (E.A.-G.); noefernandez@ub.edu (N.F.-C.); erojofra20@alumnes.ub.edu (E.R.-F.); l.pineda@ub.edu (L.P.-C.); 2 Centro de Investigación Biomédica en Red de Enfermedades Raras (CIBERER), Instituto de Salud Carlos III, 28029 Madrid, Spain; 3 Institut de Biomedicina de la Universitat de Barcelona (IBUB), 08028 Barcelona, Spain; estefaniamoreno@ub.edu (E.M.); vcasado@ub.edu (V.C.); 4 Institut de Recerca Sant Joan de Déu (IR-SJD), 08950 Esplugues de Llobregat, Spain; 5 Centre for Neuropsychiatric Disorders, Department of Biomedicine, University of Bergen, N5009 Bergen, Norway; Sadaf.Ghorbani@uib.no (S.G.); Rune.Kleppe@uib.no (R.K.); Jan.Haavik@uib.no (J.H.); 6 Child and Adolescent Mental Health Unit, Hospital Universitari Mútua de Terrassa, 08221 Terrassa, Spain; ahervas@mutuaterrassa.cat (A.H.); irueda@sjdhospitalbarcelona.org (I.R.); 7 IGAIN, Global Institute of Integral Attention to Neurodevelopment, 08007 Barcelona, Spain; 8 Department of Biochemistry and Molecular Biomedicine, Faculty of Biology, University of Barcelona, 08028 Barcelona, Spain; 9 Neuroscience Research Australia, Sydney, NSW 2031, Australia; j.fullerton@neura.edu.au; 10 School of Medical Sciences, University of New South Wales, Sydney, NSW 2052, Australia; 11 Department of Cognitive Neuroscience, Donders Institute for Brain, Cognition and Behaviour, Radboud University Medical Center, 6525 HR Nijmegen, The Netherlands; Jan.Buitelaar@radboudumc.nl; 12 Karakter Child and Adolescent Psychiatry University Centre, 6525 GC Nijmegen, The Netherlands; Nanda.Lambregts-Rommelse@radboudumc.nl; 13 Department of Psychiatry, Donders Institute for Brain, Cognition and Behaviour, Radboud University Medical Center, 6525 HR Nijmegen, The Netherlands; Barbara.Franke@radboudumc.nl; 14 Department of Human Genetics, Donders Institute for Brain, Cognition and Behaviour, Radboud University Medical Center, 6525 HR Nijmegen, The Netherlands; 15 Department of Psychiatry, Psychosomatic Medicine and Psychotherapy, University Hospital Frankfurt, 60590 Frankfurt am Main, Germany; andreas.reif@kgu.de; 16 Department of Child and Adolescent Psychiatry, Psychosomatics and Psychotherapy, Autism Research and Intervention Center of Excellence Frankfurt, JW Goethe University, 60323 Frankfurt am Main, Germany; andreas.chiocchetti@kgu.de (A.G.C.); christinemargarete.freitag@kgu.de (C.F.); 17 Division of Psychiatry, Haukeland University Hospital, 5021 Bergen, Norway; 18 Centro de Biología Molecular “Severo Ochoa”, Universidad Autónoma de Madrid/CSIC, C/Nicolás Cabrera, 1, Campus UAM, 28049 Madrid, Spain

**Keywords:** autism, 14-3-3 gene family, rare variants, common variants, transcriptomics, schizophrenia, *YWHAZ*, *YWHAE*

## Abstract

The 14-3-3 protein family are molecular chaperones involved in several biological functions and neurological diseases. We previously pinpointed *YWHAZ* (encoding 14-3-3ζ) as a candidate gene for autism spectrum disorder (ASD) through a whole-exome sequencing study, which identified a frameshift variant within the gene (c.659-660insT, p.L220Ffs*18). Here, we explored the contribution of the seven human 14-3-3 family members in ASD and other psychiatric disorders by investigating the: (i) functional impact of the 14-3-3ζ mutation p.L220Ffs*18 by assessing solubility, target binding and dimerization; (ii) contribution of common risk variants in 14-3-3 genes to ASD and additional psychiatric disorders; (iii) burden of rare variants in ASD and schizophrenia; and iv) 14-3-3 gene expression using ASD and schizophrenia transcriptomic data. We found that the mutant 14-3-3ζ protein had decreased solubility and lost its ability to form heterodimers and bind to its target tyrosine hydroxylase. Gene-based analyses using publicly available datasets revealed that common variants in *YWHAE* contribute to schizophrenia (*p* = 6.6 × 10^−7^), whereas ultra-rare variants were found enriched in ASD across the 14-3-3 genes (*p* = 0.017) and in schizophrenia for *YWHAZ* (meta-*p* = 0.017). Furthermore, expression of 14-3-3 genes was altered in post-mortem brains of ASD and schizophrenia patients. Our study supports a role for the 14-3-3 family in ASD and schizophrenia.

## 1. Introduction

Autism spectrum disorder (ASD) is characterized by impairments in communication and social interactions, and the presence of repetitive and restrictive behaviours [1]. However, the clinical picture is often accompanied by additional features, such as intellectual disability (ID), epilepsy, language impairment, anxiety, sleep disorders, and attention-deficit hyperactivity disorder (ADHD) [2]. ASD has a prevalence of approximately 1.5% in the general population [3,4] and large studies suggest an unequivocal genetic contribution to its aetiology. Indeed, family and twin studies indicate a heritability of around 80%, which represents one of the highest amongst neuropsychiatric disorders [5,6,7]. Despite the substantial role of genetic factors in the disorder, the genetic architecture is not fully dissected and many of the underlying genes are yet to be identified. Also, the genetic relationships amongst comorbid phenotypes remain largely unknown. 

Genetic studies suggest a multi-hit model of inheritance in which a combination of risk alleles, including both common variants of small effect size and rare variants with higher penetrance, contribute to the phenotype [8,9]. Despite the large number of association studies in the last decade, the robust identification of common risk alleles for ASD has been elusive, and the first genome-wide association studies (GWAS) performed with reasonably large samples [10,11,12] pinpointed associations with single nucleotide polymorphisms (SNPs) that were subsequently not replicated in a large European sample [13]. A GWAS recently performed in 18,381 ASD patients and 27,969 controls identified five genome-wide significant loci [14], suggesting the need of large samples to identify common risk variants. Regarding rare variants in ASD, the first whole-exome sequencing (WES) studies focused on de novo variants and suggested a substantial role for this class of damaging mutations in the aetiology of the disorder [15,16,17]. These approaches were crucial to pinpoint novel genes involved in the disorder [18], and recently the largest WES study reported 102 ASD risk genes in a sample of 11,896 cases [19]. WES studies were also performed to address the impact of rare inherited variants [18,20,21,22]. We performed the first WES study in multiplex families with autism that suggested a role for genome-wide truncating mutations in the aetiology of ASD [22]. A current genetic model would implicate a higher number of gene-disrupting variants in severe ASD phenotypes, increasing symptom severity and comorbidity with ID [2,19]. 

In our first WES study [22] we identified a truncating mutation in the *YWHAZ* gene (c.659-660insT, p.L220Ffs*18) that was transmitted from a mother with depression to an ASD sib-pair. In that previous study, the *YWHAZ* gene was the main interconnected node in a network including mutated genes identified by previous WES studies and other genes implicated in ASD. This gene encodes 14-3-3ζ, a protein involved in a range of biological processes including cell cycle, transcription, neuronal development, migration and neurite outgrowth [23,24,25]. 14-3-3ζ is one of the seven members (β, γ, ε, η, ζ, σ, θ) of the highly conserved 14-3-3 protein family. These proteins exert their function as homo- and heterodimers through protein-protein interaction with a wide range of target proteins, typically binding to phosphorylated serine and threonine residues [26]. Several studies have demonstrated the role of *YWHAZ* and other family members in neurogenesis and neurodifferentiation, and its possible implication in neurodevelopmental disorders [24,27]. Recent studies suggested that decreased 14-3-3ζ protein levels in ASD may be responsible for deficits in melatonin synthesis observed in ASD via the downregulation of the aralkylamine N-acetyltransferase (AANAT) and acetylserotonin o-methyltransferase (ASMT) enzymatic activity [28,29,30]. Moreover, *YWHAZ* knock-out mice show impaired cortical development and larger lateral ventricles, reduced dendritic and synaptic density, aberrant neuronal migration in hippocampus, abnormal mossy fibers connectivity, and cognitive deficits [31,32,33]. 

Interestingly, several members of the 14-3-3 gene family have been implicated in psychiatric disorders, including reported associations of *YWHAB*, *YWHAE*, *YWHAZ* and *YWHAH* with schizophrenia [34,35,36,37,38,39]; *YWHAE* and *YWHAQ* with ADHD [39]; *YWHAH*, *YWHAG* and *YWHAE* with bipolar disorder [39,40]; *YWHAE* and *YWHAQ* with major depressive disorder [39,41]; and *YWHAE* with suicide attempts [42]. However, most of these studies were performed in small samples and lacked replication in larger populations. Furthermore, altered levels of 14-3-3 proteins were found in the blood or brains of patients with ASD [29,43,44], schizophrenia [45,46,47,48] and bipolar disorder [48]. Interestingly, microduplications of *YWHAE,* which encodes 14-3-3ε that form stable heterodimers with 14-3-3ζ, were reported in ASD patients; whereas microdeletions involving both *YWHAE* and *PAFAH1B1* genes cause Miller–Dieker syndrome, a form of lissencephaly with ID and seizures [49,50,51,52,53], and deletions including *YWHAG* and *HIP1* were related to epilepsy, learning problems and ID [54].

Animal models deficient for 14-3-3 proteins show a variety of behavioural manifestations related to psychiatric disorders: *Ywhaz* (14-3-3ζ) deficient mice present hyperactivity, impaired memory, lower anxiety and impaired sensorimotor gating [32,33]; *Ywhae* (14-3-3ε) deficient mice present enhanced anxiety-like behaviour, defects in working memory, increased locomotor activity and sociability [34,55]; and heterozygous knock-out mice deficient for *Ywhag* (14-3-3γ) show hyperactivity and depressive-like behaviour [56]. Furthermore, a recent study reported that the specific inhibition of 14-3-3 proteins in the hippocampus of mice is sufficient to cause hyperactivity, reduce sensorimotor gating and impair associative learning and memory [57].

Based on these aforementioned reports, we hypothesize that the 14-3-3 gene family may play an important role in the susceptibility to psychiatric disorders. Thus, here we aim to: (i) gain molecular insight for the role of the *YWHAZ* truncating mutation p.L220Ffs*18 previously reported in a family with distinct psychiatric disorders; (ii) assess the contribution of common and rare variants of the 14-3-3 gene family (*SFN*, *YWHAB*, *YWHAE*, *YWHAG*, *YWHAH*, *YWHAQ* and *YWHAZ*) to ASD and other psychiatric disorders; and (iii) explore possible altered expression levels of this gene family in psychiatric disorders. 

## 2. Experimental Section

### 2.1. Expression, Purification and Solubility Testing of Recombinant 14-3-3ζ Wild-Type and Mutated Proteins

The recombinant human YWHAZ wild-type (WT) and mutant were expressed in *E. coli* using pGEX-2TK expression system, and purification of the soluble fractions as fusion proteins with glutathione-S-transferase (GST) was performed according to previous protocols [58], see also supplementary information for details. The 14-3-3ζ mutated protein replicating the C-terminal amino acid alteration from the p.L220Ffs*18 insertion mutation, was generated using site-directed mutagenesis as detailed in the supplementary information. 

Due to low solubility of the GST-14-3-3ζ_mut protein we tested expression and solubility at different temperatures (induction time, h) of 30 (4 h), 25 (5 h) and 20 (6 h) °C and compared total and soluble lysate to that of WT on sodium dodecyl sulphate-polyacrylamide gel electrophoresis (SDS-PAGE, see supplementary information for details).

### 2.2. Functional Assessment of 14-3-3ζ WT and Mutant Proteins by Surface Plasmon Resonance

One of the best-characterized binding targets of 14-3-3 proteins is tyrosine hydroxylase (TH) phosphorylated at serine 19 (THpSer19) [58,59]. Human TH was purified and phosphorylated on serine 19 using p38 regulated/activated protein kinase (PRAK, also referred to as MK2) as previously described [60]. The WT and mutated 14-3-3ζ proteins were assessed for their binding to THpS19 or non-phosphorylated TH using surface plasmon resonance (Biacore 3000, Cytiva, Marlborough, MA, USA) as previously described [58,60]. GST-14-3-3 proteins were immobilized on CM5 sensor chips according to manufacturer’s instructions using the GST-capture kit (Cytiva) giving similar amounts of immobilized GST-14-3-3ζ_WT and GST-14-3-3ζ_mut. Target protein binding was assessed at 25 °C, using the HBS-P Buffer provided by the manufacturer (10 mM HEPES pH 7.4, 150 mM NaCl and 0.005% polysorbate 20). Different concentrations of Ser19 phosphorylated TH were injected multiple times for multiple immobilizations. We used the unphosphorylated TH as a negative control of the binding. The resulting sensograms were analyzed with the BIAevaluation v3.2 software (Biacore AB, Uppsala, Sweden).

### 2.3. Assessing the Dimerization of Mutant and Wild-Type Proteins (14-3-3ζ and 14-3-3σ) in Cells by Bioluminescence Resonance Energy Transfer (BRET) Assay

Plasmids were obtained for expressing fusion proteins of different 14-3-3 members (14-3-3ζ, 14-3-3ε, 14-3-3σ) with Rluc (Renilla luciferase, donor) and EYFP (enhanced yellow variant of GFP, acceptor), as described in the supplementary information. The ability of mutant 14-3-3ζ to form heterodimers with 14-3-3ε, and of mutant 14-3-3σ to form homodimers, was assessed using a bioluminescence resonance energy transfer (BRET) assay. 

Human embryonic kidney cells (HEK-293T) were grown in Dulbecco’s modified Eagle’s medium (DMEM) supplemented with 10% fetal bovine serum, 100 U/mL penicillin and 100 μg/mL streptomycin (GIBCO, Carlsbad, CA, USA), in a 5% CO_2_ humidified atmosphere at 37 °C. The cell line was grown in 6-well plates (35-mm-diameter wells) at a density of 5.0 × 10^5^ cells/well for transfection using CaCl_2_ as described in the supplementary information. Cells were co-transfected with the cDNA construct coding for Rluc-target-protein-1, acting as BRET donor, and increasing amounts of the cDNA construct coding for YFP-target-protein-2 as BRET acceptor (see Table S1 for the amounts of cDNA used). D(1A) dopamine receptor (DRD1) fusion protein with YFP or Rluc [61] was used as a negative control of the dimerization, and co-transfected with the corresponding tested constructs (Table S1). After 48 h upon transfection, cells were washed twice with Hanks’ balanced salt solution pH 7.4 (HBSS, 137 mM NaCl, 5 mM KCl, 1.26 mM CaCl_2_, 0.4 mM MgSO_4_, 0.5 mM MgCl_2_, 0.34 mM Na_2_HPO_4_, 0.44 mM KH_2_PO_4_, 10 mM HEPES) supplemented with 1% glucose (*w*/*v*), detached and resuspended in the same buffer. Protein concentration was determined using the Bradford assay kit (Bio-Rad, Munich, Germany) and all cell suspensions were diluted with HBSS to obtain a final concentration of 0.2 mg/mL of protein.

In order to quantify fluorescence, cell suspensions (20 μg of protein) were distributed in duplicates in a 96-well black microplate with a transparent bottom (Porvair, King’s Lynn, UK). Fluorescence was then measured using a FLUOstar Optima fluorimeter (BMG Labtechnologies, Offenburg, Germany) equipped with a high-energy xenon flash lamp, and a 10 nm bandwidth excitation filter at 400 nm reading. A PHERAstar Flagship FSX fluorimeter (BMG Labtechnologies, Offenburg, Germany) was used for BRET and luminescence measurements. Cell suspensions (20 μg of protein) were distributed in duplicates in a 96-well white opaque microplate (Porvair, King’s Lynn, UK) and coelenterazine H (Molecular Probes Europe, Leiden, The Netherlands) was added at a final concentration of 5 mM. One minute after adding coelenterazine H, luminescence readings were collected using sequential integration of signals detected at 440–500 nm and 510–590 nm. Luminescence measurements of the same samples were performed after 10 min of incubation with coelenterazine H. Cells expressing BRET donors alone were used to determine background. The BRET ratio is defined as: ((emission at 510–590 nm)/(emission at 440–500 nm))–Cf; where Cf corresponds to (emission at 510–590 nm)/(emission at 440–500 nm) for the donor construct expressed alone in the same experiment. Data were fitted to a non-linear regression equation, assuming a single phase saturation curve with GraphPad Prism software (San Diego, CA, USA).

### 2.4. Common Variants in the 14-3-3 Family in Autism Spectrum Disorder (ASD): Association Study in Our Sample

#### 2.4.1. Subjects of Our ASD Cohorts 

The cohort used in the case-control association study consisted of 727 ASD patients and 714 gender-matched controls from three European populations: Spanish, Dutch and German (Table S2). The cohort used for mutational screening employed a subset of 288 ASD patients from the same three populations (Table S2). All individuals had European ancestry. ASD patients were assessed using the ADI-R (Autism-Diagnostic Interview-Revised) [62] and, where possible, also the ADOS (Autism Diagnostic Observation Schedule) [63]. Cytogenetic abnormalities or a positive Fragile X test were considered exclusion criteria. The study was approved by the relevant ethics committee from each center and written informed consent was obtained from all parents/guardians or, where possible, by affected individuals, according to the Helsinki Declaration. Genomic DNA was extracted from peripheral blood samples using the standard salting-out method [64].

#### 2.4.2. Common Variant Association Study of the 14-3-3 Family in Our ASD Sample

SNP selection was performed to encompass common genetic variants across the seven genes of the 14-3-3 family (*SFN*, *YWHAQ*, *YWHAG*, *YWHAZ*, *YWHAB*, *YWHAH* and *YWHAE*). Each gene included a 5kb flanking region (both 5′ and 3′), and patterns of linkage disequilibrium (LD) were considered using the Central European (CEU) panel of HapMap project data (www.hapmap.org; phases 1, 2, 3; release 28). A total of 42 tagSNPs were selected using the Tagger implementation in HaploView v4.2 [65], according to the following criteria: r^2^ ≤ 0.8 and minor allele frequency (MAF) ≥ 0.05. The sample of 1441 subjects was genotyped using iPlex-Sequenom technology (Sequenom, San Diego, CA, USA) at the Spanish National Genotyping Center (CeGen). Duplicates were included as controls for genotyping quality. After quality control procedures, 36 individuals with genotyping rate lower than 90% were removed from the study, setting the final sample to 1405 individuals (713 cases and 692 controls). From the 42 SNPs initially genotyped, four assays failed, and one SNP was monomorphic. The genotyping rate for the 37 remaining SNPs was 99.2%. One of the SNPs was excluded for quality reasons, but all the remaining 36 SNPs were in Hardy–Weinberg equilibrium (threshold set at *p* < 0.01 in controls). Thirty-four SNPs showed a MAF > 0.05 in our sample and were examined for association with ASD (Table S3). LD patterns and D’ values were determined in our sample data with Haploview v4.2 [65] (Figure S1). A case-control association study under the additive model was run with the PLINK package [66]. 

### 2.5. Common Variant Associations with the 14-3-3 Gene Family across Psychiatric Disorders Using Public Genome-Wide Association Studies (GWAS) Data

We assessed the contribution of common variation in the seven 14-3-3 genes to psychiatric disorders using GWAS summary statistics from the Psychiatric Genomics Consortium (PGC), Broad Antisocial Behaviour Consortium (BroadABC) and Integrative Psychiatric Research Consortium (iPSYCH). We considered the following phenotypes: attention-deficit/hyperactivity disorder (ADHD) [67], anti-social behaviour (ASB) [68], anxiety [69], autism spectrum disorder (ASD) [14], bipolar disorder (BD) [70], major depressive disorder (MDD) [71], obsessive-compulsive disorder (OCD) [72], schizophrenia [73] and cross-disorder meta-analysis [74] (see details in Table S4). All summary statistics used for subsequent analysis had a MAF ≥ 0.01 and info-score for imputation quality ≥ 0.6. A gene-based association study was performed with MAGMA (v1.06) [75] using the 1000 Genomes Project Phase 3 (Build 37/European data only) as a reference panel. We also performed a self-contained gene-set analysis considering the whole 14-3-3 gene family for each of the eight psychiatric phenotypes and for the cross-disorder meta-analysis.

### 2.6. Rare Variant Analysis of the 14-3-3 Gene Family: Mutational Screening in Our ASD Sample

The seven genes of the 14-3-3 family were analyzed in 288 Caucasian ASD patients by high-throughput sequencing using the Ion Torrent platform (ThermoFisher Scientific, Waltham, MA, USA) at the Centre for Research in Agricultural Genomics (CRAG). A total of 57 tagged-primer pairs were designed with the Ion Ampliseq Designer (ThermoFisher Scientific) and covered 96.3% of all coding exons across the 14-3-3 gene family (Table S5), including the splice sites and part of the 5′ and 3′ untranslated regions (UTR) (Figure S2). The corresponding amplicons were sequenced in 288 ASD patients (182 Spanish, 94 Dutch, 12 Germans), including the MT_160.3 patient, heterozygous carrier for the previously described c.659_660insT mutation leading to p.L220Ffs*18 in the *YWHAZ* gene [22], as a positive control. Genomic DNA was pooled in groups of three subjects to minimize costs. The results were processed using the Ion Reporter Software (ThermoFisher Scientific) under somatic variant calling parameters to identify low-frequency variant calls (average read depth 1400X), and the function Variant Analysis of the Ingenuity Pathway Analysis software (http://www.ingenuity.com/products/ipa) was employed to assess the identification and the molecular nature of the variants identified in our sample. To handle analysis of pooled sequencing, every single change identified in a pool was subjected to Sanger validation in the three subjects present in the reaction, in order to confirm the variant and determine the carrier status of each individual.

### 2.7. Rare Variant Analysis of the 14-3-3 Gene Family: ASD and Schizophrenia Public Datasets

The impact of rare variants across the seven genes of the 14-3-3 family was assessed and further extended using publicly available sequencing data of schizophrenia, ASD and control cohorts from the following sources: (i) whole-exome sequencing (WES) from the Sweden-Schizophrenia population-based case-control (database of Genotypes and Phenotypes (dbGAP) accession: phs000473.v2.p2) (6135 cases and 6245 controls); (ii) ARRA Autism Sequencing Collaboration (dbGAP accession: phs000298.v3.p2) (1288 unrelated ASD probands); (iii) European ASD samples, which included our previously sequenced sample of 288 ASD patients plus 348 additional ASD patients from Germany; (iv) Medical Genome Reference Bank (2845 healthy Caucasian Australians aged > 75). The selection of variants was based on: (1) their predicted pathogenicity using the Variant Effect Predictor annotation tool software (https://www.ensembl.org/Tools/VEP): missense mutations predicted to be damaging in both SIFT (Sorting Intolerant From Tolerant) and Polyphen2 (Polymorphism Phenotyping v2)), and with CADD (Combined Annotation Dependent Depletion) > 20 for canonical splice site variants, stop codon mutations and indels leading to frameshift; and (2) minor allele frequency (MAF < 0.0001) in non-Finnish European populations from the Genome Aggregation Database (http://gnomad.broadinstitute.org/) as previously described [18]. A chi-square statistic was used to compare the schizophrenia patient sample (6135 cases) and combined ASD datasets (1924 cases) with the combined control datasets (9090 individuals). Additionally, we explored the impact of rare variants for each of the 14-3-3 genes as reported by the Autism Sequencing Consortium (ASC) (https://asc.broadinstitute.org/) and the Schizophrenia Exome-sequencing Meta-Analysis (SCHEMA) (https://schema.broadinstitute.org/). ASC represents the largest source of data of rare variants for genetic studies of ASD, which includes de novo variant calls for 6430 probands and 2179 unaffected siblings (family-based dataset), and rare variants identified in 5556 ASD patients and 8809 controls (case-control dataset). Similarly, SCHEMA combines data for rare variants across several world-wide populations with a joint sample of 24,248 schizophrenia patients and 97,322 controls (case-control dataset), and 3444 schizophrenia trios (family-based dataset).

### 2.8. Expression of the 14-3-3 Genes in Autism Spectrum Disorder and Schizophrenia

Differential expression of the seven 14-3-3 genes was assessed using transcriptomic data from post-mortem brain regions of ASD and schizophrenia patients in publicly available human datasets, either in the Gene Expression Omnibus (GEO, http://www.ncbi.nlm.nih.gov/geo) or in published articles. We found 39 studies with available information on gene expression in brain: 12 in ASD patients and 27 in schizophrenia patients. In particular, in the case of ASD we analysed data on altered gene expression in brain from 11 papers and one GEO dataset (PubMed ID: 27919067, 29859039, 21614001, 22457638, 22984548, 18006270, 18378158, 25494366, 27219343, 27685936, 30545856 and GSE38322). For schizophrenia we analysed data on brain gene expression from 14 papers (30545856, 25113377, 24287731, 21091092, 24167345, 23904455, 24686180, 24886351, 22031440, 18778695, 26818902, 22212594, 22954356, 29931221) and 13 GEO datasets (GSE46509, GSE37981, GSE21935, GSE21138, GSE17612, GSE12654, GSE87610, GSE53987, GSE62191, GSE35977, GSE12649, GSE12679, GSE35978). Differential expression was assessed in multiple brain areas, including hippocampus, cerebellum or cortex, depending on the dataset.

## 3. Results

### 3.1. Functional Effect of the YWHAZ (14-3-3ζ) Mutation p.L220Ffs*18

**Figure 1:**
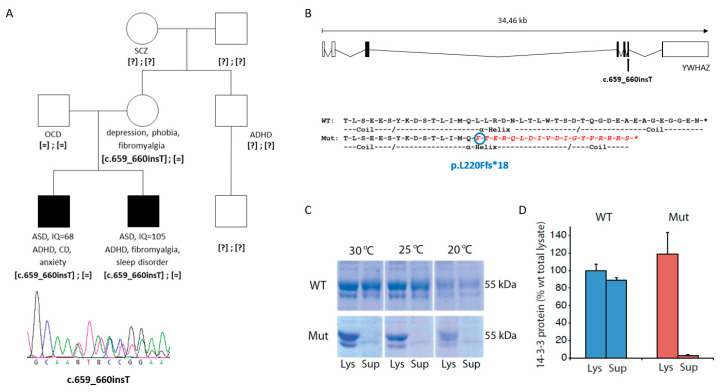
Mutation identified in *YWHAZ* in two siblings with ASD. (**A**) Pedigree of the family carrying the c.659-660insT mutation in *YWHAZ* and below the Sanger sequence of the truncating variant. Abbreviations: ADHD, attention-deficit hyperactivity disorder; ASD, autism spectrum disorder; CD, conduct disorder; IQ, intelligence quotient; OCD, obsessive-compulsive disorder; SCZ, schizophrenia. [=], wild-type allele; [?], unknown genotype. (**B**) Location of the mutation in the *YWHAZ* gene, and comparison of the last amino acids of the wild-type (WT) and mutant (Mut) 14-3-3ζ protein, showing in red the amino acids that diverge in the mutant protein. (**C**) Coomassie stained sodium dodecyl sulphate-polyacrylamide gel electrophoresis (SDS-PAGE) of glutathione-S-transferase (GST)-14-3-3ζ (WT and mutant) expressed in *E. coli* strain at different temperatures. Aliquots of the bacteria were lysed, and the amount of soluble protein was assessed by comparing total lysate (Lys) to supernatant after centrifugation of the lysate (Sup) for wild-type 14-3-3ζ (WT) and mutant 14-3-3ζ (Mut). (**D**) Quantification of solubility by measuring the major GST-14-3-3 band (55 kDa) for WT and Mut expressed at 30 °C. Data presented as means and error bars denote the standard deviation (*n* = 3; *p* = 1.7 × 10^−3^ for Mut Lys vs. Mut Sup; *p* = 1.4 × 10^−4^ for WT Sup vs. Mut Sup; t-test, two sided).

We firstly aimed to assess the functional consequences of a truncating variant (c.659_660insT, p.L220Ffs*18) previously identified in the *YWHAZ* gene and transmitted from a mother with depression, phobia and fibromyalgia to two siblings both with ASD and attention-deficit hyperactivity disorder (ADHD) [22]. The maternal grandmother was diagnosed with schizophrenia (Figure 1A), although DNA from this case was unfortunately not available for mutation analysis. 

This truncating variant does not meet criteria to trigger degradation of the corresponding mRNA by nonsense-mediated RNA decay (NMD), since it is located 22 nucleotides before the last exon-exon junction (Figure 1B) [76,77]. Therefore, we performed experiments to investigate the possible functional effect of p.L220Ffs*18 on the protein by assessing: (i) its solubility; (ii) its ability to bind tyrosine hydroxylase (TH), one of its natural targets when phosphorylated at its 14-3-3 binding site (THpSer19); and (iii) its capacity to form heterodimers with 14-3-3ε.

The mutated 14-3-3ζ protein (p.L220Ffs*18) showed decreased solubility compared to the WT 14-3-3ζ when it was expressed in prokaryotes (*E. coli*) (Figure 1C,D). The solubility did not show any improvement at different temperatures for the mutated protein (Figure 1C). The fraction of soluble mutated protein was lower than 5% (compared to 90% for the WT protein), with the vast majority remaining insoluble in the lysate fraction (Figure 1D). 

**Figure 2:**
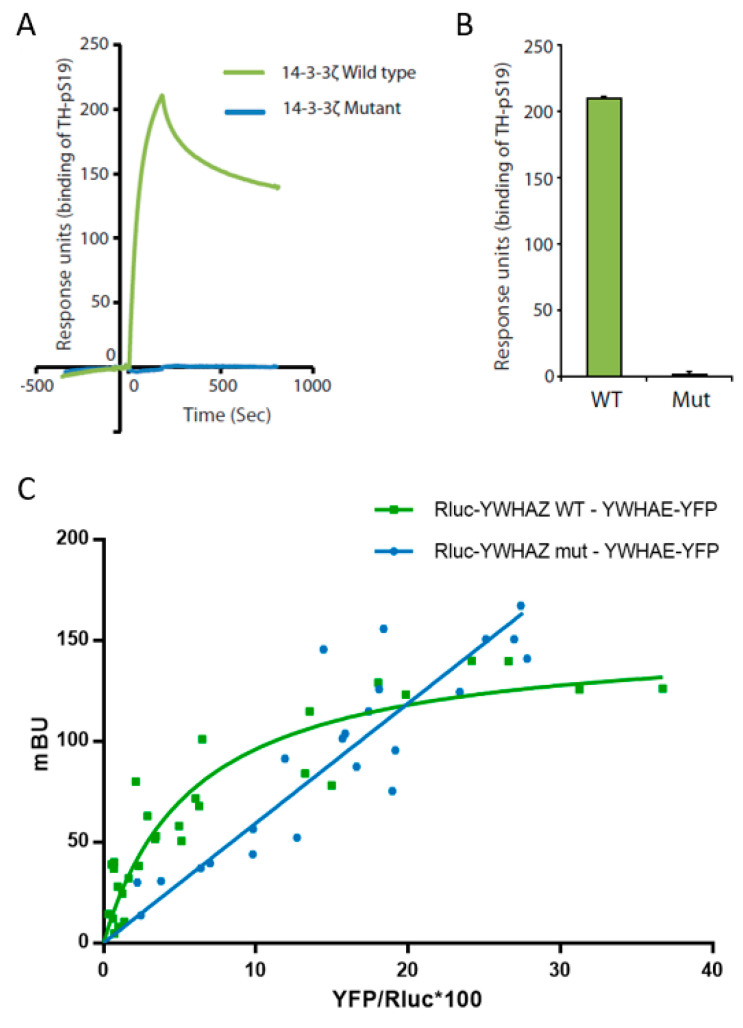
Characterization of mutant 14-3-3ζ binding capacity. (**A**) Binding of Ser19 phosphorylated human tyrosine hydroxylase (25 nM) to immobilized wild-type GST-14-3-3ζ (green) or mutant GST-14-3-3ζ (blue) using surface plasmon resonance (Biacore 3000, see Materials and Methods for details). The proteins were expressed in the BL21 Codon Plus *E. coli* strain and purified prior to the analysis. (**B**) The binding response for Ser19 phosphorylated tyrosine hydroxylase (25 nM) at the end of the injection was compared between 14-3-3ζ WT or Mut and Ser19 phosphorylated tyrosine hydroxylase (TH-pS19) (25 nM) repeated by several immobilizations and injections. Data presented as means and error bars denote the standard deviation (*n* = 3, *p* = 1.61 × 10^−8^ WT vs. Mut, two-sided t-test). (**C**) Characterization of wild-type 14-3-3ζ (YWHAZ WT) and mutant 14-3-3ζ (YWHAZ mut) interaction with 14-3-3ε (YWHAE) using a bioluminescence resonance energy transfer (BRET) assay. Rluc-YWHAZ WT co-transfection with an increasing amount of YWHAE-YFP gives a saturable positive signal (green), whereas the signal obtained co-transfecting Rluc-YWHAZ mut with an increasing amount of YWHAE-YFP fits with a linear regression (blue). mBU, BRET ratio expressed in milli-BRET units. The relative amount of BRET is given as a function of YFP/Rluc*100, where YFP corresponds to the fluorescence signal due to the increasing amount of donor and Rluc corresponds to the stable luminescence signal measured at 10 min. Values shown correspond to independent experiments (*n* = 4).

The 14-3-3 proteins exert their function as homo- or heterodimers through binding to their target proteins, usually in a Ser/Thr phosphorylation-dependent manner [78]. We assessed the ability of 14-3-3ζ p.L220Ffs*18 to bind tyrosine hydroxylase (TH), the rate limiting enzyme in the synthesis of dopamine and one of its canonical target proteins. WT 14-3-3ζ has previously been reported to bind with nM affinity to Ser19-phosphorylated TH (THpSer19) (THpSer19) [58,79]. By using surface plasmon resonance, we found that the mutant protein expressed in prokaryotes (*E. coli*) lost its ability to bind the THpSer19 compared to the WT protein (Figure 2A,B).

We further assessed the capacity of both 14-3-3ζ_WT and 14-3-3ζ_mut proteins to interact with 14-3-3ε and form heterodimers, all expressed in a human cell line (HEK 293T cells). Through BRET assays we were able to detect the interaction between WT 14-3-3ζ and 14-3-3ε, as shown by a positive and saturable BRET signal (Figure 2C). Our results showed that the mutant 14-3-3ζ lost its capacity to form heterodimers with 14-3-3ε, since the linear relationship with the acceptor/donor ratio (YFP/Rluc) suggested lack of interaction (Figure 2C). We used DRD1, not known to be a 14-3-3 target, fused either to luciferase or to yellow fluorescent protein (DRD1-Rluc and DRD1-YFP) as negative controls. No interactions were identified, obtaining linear non-specific BRET signals, confirming the specificity of the WT 14-3-3ζ and 14-3-3ε interaction (Figure S3). Remarkably, when expressed in HEK293T cells for BRET assays, the 14-3-3ζ mutant protein only showed a detectable signal when transfecting at least 10 times the amount of plasmid required for the WT (Table S1), indicating likely protein degradation, in line with the decreased solubility observed in *E. coli*.

Thus, a damaging effect of the truncating mutation p.L220Ffs*18 on 14-3-3ζ was confirmed, with loss of function of the altered protein.

### 3.2. Common Variants Across the 14-3-3 Gene Family in ASD and Other Psychiatric Disorders 

We sought for common genetic risk variants in the gene family encoding the 14-3-3 proteins in ASD and in other psychiatric disorders.

A case-control study was first performed in our sample of 713 ASD patients and 692 controls, both with European ancestry, investigating the common genetic variability of the 14-3-3 gene family (*SFN*, *YWHAB*, *YWHAE*, *YWHAG*, *YWHAH*, *YWHAQ* and *YWHAZ*) tagged by 34 SNPs. Only the variant rs1883660, located at the 3′UTR of the *SFN* gene, showed a nominal association with ASD (*p* = 0.01), but it did not remain significant after Bonferroni correction for multiple testing (Table S3). 

We further extended the analysis for contribution of common variants in the seven genes of the 14-3-3 family to large GWAS datasets of eight psychiatric disorders, using summary statistics from the PGC, BroadABC and iPSYCH GWAS datasets (Table S4). The gene-based association study showed nominal associations for four out of seven genes with several psychiatric disorders: *YWHAB* with ADHD and schizophrenia, *YWHAE* with bipolar disorder and schizophrenia, *YWHAZ* with MDD and schizophrenia, and *SFN* with anxiety (Table 1).

However, the association found between *YWHAE* and schizophrenia (*p* = 1.35 × 10^−6^; 33,640 cases and 43,456 controls) was the only surviving Bonferroni correction for 7 genes and 9 phenotypes. The gene-based results from the cross-disorder meta-analysis combining data across eight psychiatric disorders also showed a significant association with *YWHAE* (*p* = 1.01 × 10^−5^). We also performed a combined gene-set association analysis that indicates a nominal association of the whole 14-3-3 gene family with schizophrenia (*p* = 0.018) (Table S6).

### 3.3. Rare Variants in the 14-3-3 Gene Family in ASD and Schizophrenia 

We investigated the role of rare variants in the 14-3-3 gene family in ASD and schizophrenia. First, we used next-generation sequencing to search for rare variants in the seven 14-3-3 genes in 288 ASD patients from our European collection, identifying nine rare variants (Table S7). All variants were validated by Sanger sequencing and the parental origin was assessed when possible (Table S7). Two detected variants were predicted to be deleterious and were found in the *SFN* gene in the same patient (p.E75del and p.T165S) together with a third variant predicted as benign (p.S149L). All three variants were present on the same chromosome and transmitted from the mother to an affected ASD proband (Figure S4A,B). We subsequently assessed the functional effect of 14-3-3σ carrying these three changes using BRET assays, observing that the mutant protein was still able to interact with both the WT and the mutated 14-3-3σ protein, as shown by positive saturable signals with similar BRET_50_ and BRETmax (Figure S4C). The specificity of this interaction was confirmed by negative controls, obtaining linear non-specific BRET signals (Figure S4D). These results suggest that these rare variants do not impact dimerization.

We also explored the impact of ultra-rare variants (URV) across the 14-3-3 gene family in an extended sample of ASD patients, as well as in a sample of schizophrenia patients. For this objective we used publicly available sequencing data, which comprised 1924 ASD probands, 6135 schizophrenia patients, and 9090 control individuals. The high degree of evolutionary conservation of the seven 14-3-3 genes [80,81] and the limited sample size, resulted in relatively limited numbers of genetic variants. This prompted us to combine the data for the whole gene family. Interestingly, a significant burden of URVs was observed for ASD in the 14-3-3 family when compared to controls (7 in 1924 ASD cases vs. 11 in 9090 controls, *p* = 0.017), driven by a splice site variant in *YWHAE* (rs756213490), which was found in four unrelated ASD probands and not in controls (Table 2).

No significant burden was identified for schizophrenia (3 in 6135 cases vs. 11 in 9090 controls, *p* = 0.71). It is noteworthy that truncating variants in *YWHAZ* are reported here only in patients (i.e., the frameshift c.659_660insT found in the ASD family functionally investigated here, and a stop mutation rs754522887 found in a Swedish schizophrenia patient), and they were not observed in the control group. Furthermore, we also explored large available datasets of ASD and schizophrenia from the Broad Institute for enrichment of rare variants, and observed that when the 14-3-3 genes were considered individually, only *YWHAZ* reached significance for a higher number of SNVs in schizophrenia (meta-analysis *p* = 0.017).

### 3.4. Altered Expression of the 14-3-3 Genes in ASD and Schizophrenia

Finally, we explored potential alterations of expression of the 14-3-3 genes in post-mortem brains of ASD and schizophrenia patients using transcriptomic datasets or literature reports. From the 39 studies with available information on gene expression in the brain (12 in ASD patients and 27 in schizophrenia patients), we found 11 studies reporting significant altered expression in 14-3-3 genes (*p* < 0.05) in ASD or schizophrenia. Seven of them reported altered 14-3-3 genes expression with a false discovery rate (FDR) < 0.1.

We found altered expression of six of the seven 14-3-3 genes in at least one of the two phenotypes compared to control subjects (*p* < 0.05, FDR < 0.1) (Table 3). All six genes, except for *SFN*, showed a decreased expression in different brain areas. Interestingly, five of these genes showed alterations in expression in both ASD and schizophrenia: *YWHAB*, *YWHAE*, *YWHAH* and *YWHAZ* showed decreased expression whether *SFN* showed increased expression in both disorders. In the case of *YWHAQ*, we found decreased expression only in ASD patients compared to controls (Table 3).

## 4. Discussion

The 14-3-3 gene family encodes seven proteins that act as effectors of signaling-regulated proteins. They are highly expressed in the brain during development [82], and are involved in several neuronal processes, such as differentiation, migration, synaptogenesis, and axon guidance [24,27,31], as well as metabolic regulation [83]. Their important role in neuronal functions makes them plausible candidate genes for ASD and other psychiatric disorders. In a previous WES study we identified a truncating mutation in *YWHAZ* (encoding 14-3-3ζ), one of the members of the 14-3-3 family, present in two brothers with ASD [22]. Both individuals were diagnosed also with ADHD. One proband had mild intellectual disability (IQ of 68) and presented severe conduct disorder, aggressive behaviour and anxiety. The other proband had a normal IQ (105) and presented fibromyalgia and sleep disorder. Furthermore, in this family the mother of the ASD sib-pair, carrier of the *YWHAZ* mutation, presented with depression and other conditions, including phobia, fibromyalgia, hypothyroidism, asthma and obesity. The maternal grandmother of the ASD sib-pair was diagnosed with schizophrenia, and a maternal uncle of the two sibs had ADHD. Unfortunately, the potential segregation of this truncating variant in *YWHAZ* in the broader family could not be tested, as DNA collection was not possible. Interestingly, genes involved in dopamine neurotransmission have been reported to be associated with ADHD and ASD [84,85,86], and fibromyalgia and chronic pain have been related to decreased dopaminergic activity [87,88]. The 14-3-3 proteins act as regulators of dopamine synthesis by binding and stabilizing tyrosine hydroxylase [89]. Our study showed that the mutated protein 14-3-3ζ (p.L220Ffs*18) was not able to bind the phosphorylated tyrosine hydroxylase, a well-established molecular partner, leading likely to altered dopamine synthesis. Moreover, the mutated protein presented a decreased solubility when expressed in *E. coli* and it was not able to form heterodimers with 14-3-3ε when expressed in a human cell line. Our results are interesting also from the perspective of understanding the structural basis of 14-3-3 protein functions, as they suggest that the far C-terminal region may be involved in dimerization and phospho-target interaction, although further experiments would be needed to confirm it.

Previous research indicates that 14-3-3ζ deficient mice (*Ywhaz* knock-out) display cognitive and behavioural deficits possibly related to the dopamine system, altered hippocampal development, defective migration of pyramidal and granular neurons [33,90]. Also, the *Ywhaz* and *Ywhae* double knock-out mice show impaired neurogenesis, neuronal proliferation, differentiation and migration, and present severe seizures, indicating that both 14-3-3ζ and 14-3-3ε play a critical role during cortical development [31]. Indeed, 14-3-3 inhibition in certain brain regions in mice leads to impaired learning, working memory and long-term synaptic plasticity, symptoms that are associated with schizophrenia-like behaviours [91]. Altogether, these findings support an essential role for the *YWHAZ* gene in brain function and development, and together with our current report, highlight its contribution to neurodevelopmental disorders. 

Given the functional evidence of this truncating mutation in *YWHAZ*, and several studies that consistently relate the 14-3-3 gene family to behavioural deficits in animal models, we further investigated the possible contribution of common and rare variants in this gene family to psychiatric disorders. The association study in our ASD sample failed to identify SNPs associated with the disorder, although the sample size was limited considering the small effect sizes typical of common variants in psychiatric disorders [13]. However, a previous study with similar sample size of adult ADHD patients identified a significant epistatic effect between *YWHAE* and two other members of this gene family, *YWHAZ* and *YWHAQ* [39]. When extending our analyses into larger samples of the PGC, BroadABC and iPSYCH, we found that common variants were gene-based associated with several psychiatric phenotypes in four of the seven 14-3-3 genes (*SFN*, *YWHAB*, *YWHAE* and *YWHAZ*), although only the association between *YWHAE* and schizophrenia survived correction for multiple testing. In line with this result, several previous studies found an association of genetic variants in *YWHAE* with schizophrenia [34,55].

The possible implication of 14-3-3 members in schizophrenia was also suggested in animal studies, which showed that 14-3-3ε deficient mice present alterations in hippocampal and cortical structures due to defects in neurogenesis and neuronal migration [92,93]. In addition, 14-3-3ε deficient mice exhibit behavioural phenotypes, such as increased motor activity and decreased working memory and are used as schizophrenia-related models [34,55]. Furthermore, 14-3-3-mediated signaling seems to be strongly affected in schizophrenia patients [47,48], supporting the association of this protein family with the disorder. Interestingly, a polymorphism (rs28365859) in *YWHAE* associated with schizophrenia [34] correlates with differences in volumes of different brain regions in patients [94,95]. Taken together, these data support the contribution of *YWHAE* to schizophrenia and general brain development.

We also investigated the impact of rare variants in a small European sample of ASD sequencing all seven 14-3-3 genes. We identified 9 rare variants in 3 genes (*YWHAE*, *YWHAB* and *SFN*), including two rare variants in *SFN* that were predicted to be pathogenic (p.E75del and p.T165S) and were present on the same chromosome of the same patient. However, the functional characterization of the effect of these latter variants showed no effect on dimerization, although they may have other functional consequences. 

We then expanded the analysis of rare variants to larger samples using available sequencing datasets of ASD and schizophrenia, which pinpointed a plausible impact for rare variants in ASD when we combined data of all 14-3-3 genes. Interestingly, the effect of this association was driven by URVs from *YWHAE* and *YWHAZ*. Indeed, several exome sequencing studies in ASD patients found rare variants in *YWHAZ* and *YWHAG* [19,96,97]. We also explored sequencing data from the ASC and SCHEMA consortia for each of the 14-3-3 genes individually and an enrichment was observed only for SNVs in *YWHAZ* in the schizophrenia dataset.

Interestingly, both the expression and the splicing of several 14-3-3 genes are regulated by RNA-binding proteins that are relevant to ASD and other psychiatric disorders. In particular, RBFOX1 (RNA binding fox-1 homolog 1) regulates *YWHAE*, *YWHAG*, *YWHAQ* and *YWHAZ*, whereas FMRP (Fragile X mental retardation protein) binds *YWHAG* [98,99]. Interestingly, *RBFOX1* has been reported in several ASD genetic studies [97,100,101,102,103], as well as in studies of aggressive behaviour and several psychiatric disorders [74,104,105,106]. Also, several targets of FMRP have been suggested to play a role in ASD [98,107,108].

Thus, our results indicate that combined rare variants in 14-3-3 genes may contribute to ASD, and that common variants from 14-3-3 family members significantly associate with schizophrenia. Also, when 14-3-3 genes are considered individually, rare variants in the *YWHAZ* gene are shown to contribute to schizophrenia.

Finally, we have systematically gathered and analysed previous transcriptomic data that suggest significant alteration of the expression of 14-3-3 genes in ASD and schizophrenia in several brain regions. Altered levels of the 14-3-3 family have previously been reported in ASD and in schizophrenia patients: 14-3-3 protein levels are diminished in platelets and pineal glands in ASD patients [29,44] and 14-3-3ζ levels are reduced in post-mortem brains of schizophrenia patients [109]. Moreover, an increased expression of *SFN* and decreased expression of *YWHAB*, *YWHAE*, *YWHAG* and *YWHAQ* mRNA have been reported in leukocytes of schizophrenia patients [45]. Our analysis of available transcriptomic data is in line with these results, as we found an altered brain expression of six of the seven 14-3-3 genes in ASD and schizophrenia patients: a robust increased expression of *SFN* in both ASD and schizophrenia (reflecting the top 9th and 36th differentially expressed gene in the transcriptome, respectively [110]; and a decreased expression of the other five 14-3-3 genes).

## 5. Conclusions

Our work combines functional studies, association studies, sequencing of a European sample, and interrogation of available genetic datasets that implicate the 14-3-3 gene family in ASD and schizophrenia, suggesting shared genetics between these two disorders. 

## Figures and Tables

**Table 1:** Gene-based association analysis of each of the seven 14-3-3 genes across several psychiatric disorders.

Gene Symbol	Entrez ID	ADHD	ASB	Anxiety	ASD	BD	MDD	OCD	SCZ	Cross-Disorder
*YWHAB*	7529	**0.024**	0.923	0.098	0.711	0.629	0.777	0.170	**0.001**	0.050
*YWHAE*	7531	0.086	0.933	0.190	0.468	**0.006**	0.154	0.262	**1.35 × 10^−6^**	**1.01 × 10^−5^**
*YWHAG*	7532	0.081	0.054	0.111	0.929	0.065	0.854	0.454	0.119	0.169
*YWHAH*	7533	0.153	0.520	0.218	0.741	0.104	0.658	0.243	0.291	0.410
*YWHAQ*	10971	0.801	0.438	0.927	0.835	0.637	0.441	0.054	0.300	0.900
*YWHAZ*	7534	0.262	0.415	0.311	0.125	0.168	**0.029**	0.771	**0.001**	0.095
*SFN*	2810	0.834	0.767	**0.045**	0.272	0.648	0.849	0.375	0.979	0.966

*p*-values were calculated using MAGMA (v1.06) software. Nominal associations are highlighted in bold. Underlined values survived Bonferroni correction for multiple testing, *p* = 7.9 × 10^−4^ (7 genes and 9 phenotypes). ADHD: attention-deficit hyperactivity disorder; ASB: antisocial behaviour; ASD: autism spectrum disorder; BD: bipolar disorder; MDD: major depression disorder; OCD: obsessive-compulsive disorder; SCZ: schizophrenia.

**Table 2:** List of ultra-rare variants (URVs) across the 14-3-3 gene family found in large public datasets.

Chr:position	Ref/Alt	Gene	Data_Set (Controls/Cases)	gnomAD NFE AF	Impact	Amino Acids	SIFT	PolyPhen-2	CADD	Existing Variation
17:1264594	T/A	YWHAE	ARRA (0/4)	2.19 × 10^−5^	splice_acceptor_variant	canonical splice site	-	-	34	rs756213490
8:101961051	C/A	YWHAZ	German_ASD (0/1)		missense_variant	A/S	deleterious low_confidence (0.03)	possibly_damaging (0.799)	26.9	rs774415799
1:27189925	GGA/-	SFN	Spanish_ASD (0/1)	2.65 × 10^−5^	inframe_deletion	SE/S	-	-	21.5	rs773116730
8:101936203	A/AT	YWHAZ	Spanish_ASD (0/1)		frameshift_variant	frameshift	-	-	-	-
2:9731521	G/A	YWHAQ	Swedish_SCZ (0/1)	8.81 × 10^−6^	stop_gained	Q/*	-	-	42	rs769768341
8:101936511	G/C	YWHAZ	Swedish_SCZ (0/1)	8.84 × 10^−6^	stop_gained	S/*	-	-	40	rs754522887
22:32352395	CAAGGTGTTT TACCTGA/C	YWHAH	Swedish_SCZ (0/1)	8.80 × 10^−6^	frameshift_variant	KVFYLK/X	-	-	35	rs759467778
1:27189840	T/C	SFN	MRGB (1/0)	2.64 × 10^−5^	missense_variant	V/A	deleterious low_confidence (0.01)	probably_damaging (0.954)	28.6	rs77608477
1:27190047	A/C	SFN	MRGB (1/0)		missense_variant	E/A	deleterious low_confidence (0)	probably_damaging (0.929)	26.5	-
1:27190145	C/T	SFN	MRGB (1/0)		missense_variant	R/W	deleterious low_confidence (0.03)	possibly_damaging (0.776)	33	-
2:9725474	G/GTGTTAGG TTGT	YWHAQ	MRGB (1/0)		frameshift_variant	frameshift	-	-	-	-
8:101961101	A/C	YWHAZ	MRGB (1/0)		missense_variant	L/R	deleterious low_confidence (0.01)	probably_damaging (0.909)	27.4	-
22:32352337	T/C	YWHAH	MRGB (1/0)	8.79 × 10^−6^	missense_variant	V/A	deleterious low_confidence (0)	probably_damaging (0.999)	27.9	rs1196036662
22:32352724	A/G	YWHAH	MRGB (2/0)		missense_variant	N/S	deleterious low_confidence (0)	probably_damaging (0.991)	26.5	-
1:27189780	T/G	SFN	MRGB (1/0)	8.80 × 10^−6^	missense_variant	M/R	deleterious low_confidence (0.01)	possibly_damaging (0.631)	27.7	rs747687239
1:27190388	CTG/C	SFN	Swedish_control (1/0)	8.81 × 10^−6^	frameshift_variant	frameshift	-	-	35	rs774524068
17:1264594	T/A	YWHAE	Swedish_control (1/0)	2.19 × 10^−5^	splice_acceptor_variant	canonical splice site	-	-	34	rs756213490

ARRA_c1, ASD cases from ARRA c1 data set (dbGAP accession: phs000298.v3.p2); German_ASD, ASD samples from Germany; MGRB, Medical Genome Reference Bank; Spanish_ASD, ASD samples from Spain; Swedish_SCZ, Sweden-Schizophrenia population-based Case-Control (dbGAP accession: phs000473.v2.p2);. All the URVs variants are selected to be rare (MAF < 0.0001 in Non-Finish European population in gnomAD, https://gnomad.broadinstitute.org/) and predicted to be pathogenic both in SIFT and, and CADD > 20.

**Table 3:** Altered expression of the 14-3-3 genes in individuals with schizophrenia or autism spectrum disorder.

Gene Symbol	Disorder	FC ^&^	*p*-Value	FDR	Probe	Tissue	Study (PMID) or GEO ID	Sample, Cases vs. Controls
*YWHAB*	SCZ	−1.06	2.51 × 10^−3^	0.10	8062880	cerebellum	GSE35978	44 SCZ vs. 50 control
*YWHAB*	SCZ	−1.41	0.001	0.02	217717_s_at *	hippocampus	GSE53987	15 SCZ vs. 18 control
*YWHAB*	ASD	N/A	N/A	1.43 × 10^−3^	N/A	cortex (BA19, BA10, BA44)	Gupta et al., 2014 (25494366)	32 ASD vs. 40 control
*YWHAE*	ASD	−1.34	0.003	0.06	ILMN_1807535	cerebellum	GSE38322	15 ASD vs. 12 control
*YWHAE*	SCZ	−1.52	1.93 × 10^−4^	0.01	210317_s_at	hippocampus	GSE53987	15 SCZ vs. 18 control
*YWHAE*	SCZ	−1.10	3.31 × 10^−4^	0.04	11753092_s_at *	DLPFC	GSE87610	65 SCZ vs. 72 control
*YWHAH*	SCZ	−1.68	6.69 × 10^−5^	0.01	201020_at	hippocampus	GSE53987	15 SCZ vs. 18 control
*YWHAH*	ASD	N/A	<0.01	<0.078	N/A	DLPFC	Liu et al., 2016 (27685936)	34 ASD vs. 40 control
*YWHAQ*	SCZ	−1.02	0.004	0.03	N/A	frontal and temporal cortex	Gandal et al., 2018 (30545856)	560 SCZ vs. 936 control
*YWHAQ*	SCZ	−1.34	2.34 × 10^−5^	0.01	200693_at *	hippocampus	GSE53987	15 SCZ vs. 18 control
*YWHAZ*	ASD	−1.61	3.01 × 10^−5^	0.01	ILMN_1669286	cerebellum	GSE38322	14 ASD vs. 12 control
*YWHAZ*	SCZ	−1.87	3.07 × 10^−5^	0.01	200641_s_at *	hippocampus	GSE53987	15 SCZ vs. 18 control
*SFN*	ASD	2.18	0.001	0.03	N/A	frontal and temporal cortex	Gandal et al., 2018 (30545856)	51 ASD vs. 936 control
*SFN*	SCZ	1.34	7.69 × 10^−5^	1.33 × 10^−3^	N/A	frontal and temporal cortex	Gandal et al., 2018 (30545856)	559 SCZ vs. 936 control
*SFN*	SCZ	1.53	0.010	0.08	33323_r_at *	hippocampus	GSE53987	15 SCZ vs. 18 control

ASD, autism spectrum disorder; SCZ, schizophrenia; FDR, false discovery rate; ^&^ FC, fold change was calculated when log2FC was provided in the study; N/A, data not available; * Genes showing significant differential expression in independent probe sets targeting different transcripts/exons of the same gene, data shown corresponding to the probe with the highest fold-change.

## Supplementary Materials

The following are available online at https://www.mdpi.com/2077-0383/9/6/1851/s1: Supplementary file containing: Additional methodological procedures; Table S1. Acceptor and donor combinations of plasmids for BRET experiments, Table S2. European ASD samples genotyped in the case-control association study and used in the mutation screening. After quality control procedures the final genotyped sample consisted of 713 ASD cases and 692 controls, Table S3. Results from the ASD case-control association study (713 ASD cases and 692 controls) with tagSNPs across the 14-3-3 gene family in the overall sample under the additive model, Table S4. Description of the summary statistics of publicly available GWAS data of eight psychiatric disorders and the corresponding cross-disorder dataset used for gene-based and gene-set analyses, Table S5. Experimental design of targeted next-generation sequencing: The coding regions of 14-3-3 genes was covered by 57 amplicons, Table S6. Gene-set association results of the 14-3-3 family set of genes on eight different psychiatric phenotypes and in the cross-disorder meta-analysis dataset, Table S7. Rare variants identified in the seven 14-3-3 family genes in 287 288 European ASD patients, Figure S1. Linkage disequilibrium values among the 37 tagSNPs analyzed in this study, calculated from the whole sample (1441 individuals with European ancestry) with the Haploview software. D’ values between all the possible SNP pairs are shown, Figure S2. The 57 amplicons used in the mutational screening are depicted in green and cover the coding regions of the 14-3-3 genes. For each of the seven genes we show the amplicon ID name, the number of overlapping amplicons per exon and the genomic region, Figure S3. Negative controls in the BRET experiments of the interaction of YWHAZ WT or YWHAZ mutant (mut) with YWHAE using D(1A) dopamine receptor as a donor (DRD1-Rluc) or an acceptor (DRD1-YFP) and adjusted to a linear regression, Figure S4. Characterization of three rare inherited *SFN* variants identified in an ASD patient.
