# Supplementary material for: Involvement of the 14-3-3 Gene Family in Autism Spectrum Disorder and Schizophrenia: Genetics, Transcriptomics and Functional Analyses
Source: J Clin Med. 2020 Jun 13;9(6):1851. doi: 10.3390/jcm9061851 (PMC7356291; doi:10.3390/jcm9061851)
Supplement: Supplementary file 1 [file jcm-09-01851-s001.pdf]

SUPPLEMENTARY FILES

INVOLVEMENT OF THE 14-3-3 GENE FAMILY IN AUTISM SPECTRUM DISORDER AND SCHIZOPHRENIA: GENETICS, TRANSCRIPTOMICS AND FUNCTIONAL ANALYSES

Bàrbara Torrico, Ester Antón-Galindo, Noèlia Fernàndez-Castillo, Eva Rojo-Francàs, Sadaf Ghorbani, Laura Pineda, Amaia Hervás, Isabel Rueda, Estefanía Moreno, Janice M. Fullerton, Vicent Casadó, Jan K. Buitelaar, Nanda Rommelse, Barbara Franke, Andreas Reif, Andreas G. Chiocchetti, Christine Freitag, Rune Kleppe, Jan Haavik, Claudio Toma, Bru Cormand

This supplementary file includes:

Additional methodological procedures

Tables S1 to S7

Figures S1 to S4

References

ADDITIONAL METHODOLOGICAL PROCEDURES

DNA constructs and site-directed mutagenesis for expression in prokaryotes

The expression vector pGEX-2TK (GE Healthcare, Little Chalfont, UK) was used to clone the cDNA of the wild-type (WT) *YWHAZ* human gene at 3' of its glutathione-s-transferase (GST) tag (GST-14-3-3ζ_WT). The mutated 14-3-3ζ form was obtained through a directed mutagenesis protocol using the *Site-Directed Mutagenesis kit* (Stratagene, La Jolla, CA, USA), inserting a thymine between the nucleotides 659 and 660 of the *YWHAZ* cDNA insert as per the observed p.L220Ffs*18 mutation (GST-14-3-3ζ_mut).

Expression and purification in prokaryotes

WT and mutated fusion proteins (GST-14-3-3ζ_WT and GST-14-3-3ζ_mut) were expressed in the *BL21 Codon Plus E. coli* strain (Stratagene) by a 4h induction at 30 ºC with 1 mM of 1-thio-β-D-galactopyranoxide (IPTG). Bacteria were lysed in a lysis buffer composed of phosphate buffered saline (PBS) 10 mM imidazole, 10 mM benzamidine, with 0.5 mg/mL lysozyme and protease inhibitor cocktail (Roche, Mannheim, Germany), using the French press. Then, the fusion proteins GST-14-3-3ζ_WT and GST-14-3-3ζ_mut were purified from the soluble fraction of the lysate through affinity chromatography using *Glutathione Sepharose 4B* (GE Healthcare). The homogeneity of the purified proteins was confirmed by SDS PAGE and quantified using theoretical absorbance at 280 nm (as the mutant has lost one Trp residue) as well as by protein staining of gels. Size exclusion chromatography was used to confirm dimeric state of soluble 14-3-3 proteins as described [1].

Solubility test of 14-3-3ζ WT and mutated proteins

Induction of the expression of the two fusion proteins with IPTG was performed at different temperatures and final induction times: 4 hours at 30 ºC, 5 hours at 25 ºC and 6 hours at 20 ºC. Cells were resuspended in the lysis buffer mentioned above and lysed by sonication. Two samples were obtained and kept in 1X sample buffer for SDS-PAGE: the total lysate sample (immediately after sonication) and the soluble sample, from the supernatant fraction after 10 min centrifugation at 13,000 *g*. Then, all samples were run on 12% denaturing polyacrylamide gels (SDS-PAGE) and patterns of WT and truncated proteins were compared.

DNA constructs for expression in eukaryotes (BRET assays)

Human cDNAs for WT or mutant *YWHAZ* were amplified from a carrier ASD proband, using sense and antisense primers harboring *Kpn*I and *Eco*RI restriction sites to clone the generated amplicons into a pcDNA3 vector (Promega, Madison, WI, USA). Subsequently, cDNAs for EYFP (enhanced yellow variant of GFP) and Rluc (*Renilla* luciferase) were amplified without their stop codons, adding *Kpn*I restriction sites at both ends of the amplicon, from pEYFP-N1 (Takara Bio Inc, Otsu, Shiga, Japan) and pRluc-N1 (PerkinElmer, Wellesley, MA, United States) vectors, respectively. The resulting fragments were subcloned in the previously generated pcDNA3_YWHAZ vectors at 5' of the *YWHAZ* insert and in-frame with its start codon. Human cDNA for *YWHAE*, cloned into pcDNA3.1 (Promega), was amplified without its stop codon using sense and antisense primers harboring *Bam*HI and *Eco*RI restriction sites. The resulting fragment was subcloned at 5' of EYFP or Rluc to be in frame with their start codons in the pEYFP-N1 and pRluc-N1 vectors respectively.

Human cDNAs for WT and mutant *SFN* were obtained from the carrier ASD proband (MT_37.3), which contained the three described variants in the same chromosome. For that, cDNAs were amplified without including the stop codon, using primers harboring *Hind*III and *Bam*HI restriction sites. All the resulting fragments were then subcloned to be in frame with pEYFP or pRluc vectors.

Briefly, we obtained ten different plasmids: YFP-YWHAZ WT, YFP-YWHAZ mut, Rluc-YWHAZ WT, Rluc-YWHAZ mut, YWHAE-YFP, YWHAE-Rluc, SFN WT-YFP, SFN mut-YFP, SFN WT-Rluc, SFN mut-Rluc.

$CaCl_2$ transfection of HEK293T cells for BRET assays

HEK293T cells were cultured at 37ºC with 5% CO_2 with DMEM supplemented with 10% fetal bovine serum, 100 U/ml penicillin and 100 μg/ml streptomycin. For transfection, the day before cells were plated in 6-well plates at a density of $5x10^5$ cells/well and media was replaced 2 hours before transfection. Individual transfection reactions were prepared in 1.5ml sterile tubes. The amount of plasmid(s) (see Supplementary Table 1) to be transfected was diluted in buffered water (HEPES 2.5mM, pH 7.3) and TE (tris, EDTA, pH 8, final concentration 0.03x) to obtain a final volume of 150μl in each sterile tube. Then, 14.9μl of $CaCl_2$ 2.5M was added to each tube while vortexing and 150μl of HBS 2x (280 mM NaCl, 50 mM HEPES, 40 mM $Na_2HPO_2.2H_2O$, pH 7.00) was subsequently added drop by drop while vortexing in order to mix well the solution. All the transfection solutions were kept at room temperature for 20 minutes and each solution was added to each well containing HEK293T. Media was replaced 17 hours after transfection.

SUPPLEMENTARY TABLES

Table S1. Acceptor and donor combinations of plasmids for BRET experiments.

Donor \ Acceptor	YWHAE-YFP	SFN WT-YFP	SFN mut-YFP	DRD1-YFP
**Rluc-YWHAZ WT**	250 / 5 - 1000	-	-	575 / 200 - 6500
**Rluc-YWHAZ mut**	2250 / 5 - 1500	-	-	4250 / 200 - 4000
**YWHAE-Rluc**	-	-	-	-
**SFN WT-Rluc**	-	2.5 / 10-180	2.5 / 10-180	9 / 100-3200
**SFN mut-Rluc**	-	-	5 / 10-180	12.5 / 200-2600
**DRD1-Rluc**	20 / 20 - 700	30 / 10 - 400	20 / 10 - 140	-

The amount of each plasmid is indicated as ng of donor / range of ng of acceptor. Donor amount is indicated as an average of the weight of plasmid used in the co-transfections in order to obtain a stable luminescence signal (around 150.000 bioluminescence units) at 10 min.

Table S2. European ASD samples genotyped in the case-control association study and used in the mutation screening. After quality control procedures the final genotyped sample consisted of 713 ASD cases and 692 controls.

	Association study		Mutation screening
	**Cases (%M)**	**Controls (%M)**	**Cases (%M)**
**Spanish**	301 (87.3)	297 (87.5)	182 (86.8)
**Dutch**	233 (76.8)	228 (77.2)	94 (79.5)
**German**	179 (90)	167 (89.2)	12 (85.7)
**Total**	713 (84.7)	692 (84.5)	288 (84.5)

%M indicates the percentage of male individuals.

Table S3. Results from the ASD case-control association study (713 ASD cases and 692 controls) with tagSNPs across the 14-3-3 gene family in the overall sample under the additive model.

Gene	Chr	tagSNP	Position GRCh37/hg19	MAF (CEU)	Alleles	MAF in our sample	HWE (unaffected)	*p*-val (additive)	Genotypes ASD (AA, AB, BB)	Genotypes Controls (AA, AB, BB)
SFN	Chr1: 27189632-27190947	rs200392321**+**	27187881	0.08	T:-	-	-	-	-	-
		rs1883660	27191411	0.128	A:T	0.1433	0.8864	0.024	12/159/541	18/183/490
YWHAQ	Chr2: 9724095-9771184	rs6734469	9735900	0.381	A:G	0.4523	0.6455	0.821	154/340/219	137/349/206
		rs13022460	9749492	0.275	T:G	0.2556	0.6179	0.707	49/256/396	48/256/376
		rs16867074	9750210	0.092	G:C	0.1082	0.6848	0.413	10/141/562	6/131/555
		rs17453675	9754407	0.242	A:G	0.1883	0.9014	0.965	28/212/473	25/211/456
		rs4668625	9765461	0.35	A:G	0.3243	0.7278	0.82	78/308/325	74/298/320
		rs13417081	9770050	0.295	G:A	0.368	0.935	0.893	100/323/290	95/321/276
		rs3762535	9773586	0.491	C:T	0.4598	0.2826	0.43	155/355/202	134/358/200
		rs4145375	9773678	0.094	A:G	0.0752	0.4093	0.782	2/105/605	5/92/594
YWHAG	Chr7: 75956107-75988342	rs2961037	75952212	0.5	G:G	0.494	0.447	0.797	171/359/183	165/357/170
		rs2961033	75964343	0.25	T:C	0.1912	0.2729	0.725	22/225/466	21/225/443
		rs10241401 #	75975427	0.057	G:A	0.02954	1	-	-	-
		rs13247572	75983524	0.083	G:A	0.07367	0.763	0.311	4/104/605	2/91/599
		rs11765693	75985373	0.196	A:G	0.2811	0.4464	0.558	63/282/368	57/268/367
YWHAZ	Chr8: 101930803-101965623	rs17365305 #	101933682	0.054	G:A	0.04128	0.6176	-	-	-
		rs4734497	101934971	0.36	T:C	0.289	0.3965	0.32	58/308/347	59/270/363
		rs17462921	101938901	0.176	G:A	0.1537	0.4824	0.287	20/169/524	15/193/484
		rs3134354	101948681	0.063	C:G	0.05836	1	0.604	3/74/636	2/80/610
		rs17366009	101957311	0.072	T:C	0.06192	0.5037	0.475	5/82/619	1/79/611
		rs3100052	101967139	0.396	G:A	0.3995	0.5808	0.694	106/351/254	116/326/250

YWHAB	Chr20: 43514239-43537175	rs6031849	43514337	0.188	G:T	0.3082	0.3325	0.327	61/302/344	63/308/314
		rs2425675	43534934	0.25	G:A	0.2897	0.1656	0.938	54/306/353	50/300/342
		rs6876	43535101	0.098	T:C	0.1322	0.5277	0.308	9/161/541	11/170/511
		rs2425678	43538152	0.438	T:C	0.402	0.1099	0.221	117/354/241	95/350/246
YWHAH	Chr22: 32340478-32353590	rs3827334	32338005	0.188	A:C	0.1601	0.4722	0.631	19/195/499	14/189/489
		rs929036	32339213	0.413	C:T	0.4477	0.5908	0.797	139/357/217	144/335/213
		rs2267172	32339782	0.064	G:A	0.07153	0.7718	1	5/92/616	4/91/597
		rs5998196	32355455	0.417	T:C	0.433	1	0.421	127/353/233	134/341/216
YWHAE	Chr17:1247833-1303556	rs11650689	1244992	0.348	C:T	0.36	0.6816	0.617	100/307/306	89/326/276
		rs9393	1248392	0.054	A:G	0.08007	1	0.316	3/101/609	5/108/579
		rs7224258	1255502	0.33	G:C	0.3085	0.6508	0.256	75/304/334	64/285/343
		rs7208041	1270562	0.241	A:G	0.2164	0.04387	0.891	38/231/444	42/217/433
		rs4790082	1278700	0.446	G:A	0.4872	0.5953	0.924	174/348/191	160/353/179
		rs17625475	1280109	0.107	G:T	0.09253	0.6231	0.188	5/132/576	6/106/580
		rs10521111	1281864	0.152	A:G	0.1357	0.03108	0.685	16/165/531	19/146/527
		rs16945811	1294614	0.078	G:A	0.07585	0.7944	0.377	2/98/613	5/101/585

Chr: chromosome; SNP: single nucleotide polymorphism; MAF: minor allele frequency in our sample; HWE: Hardy-Weinberg equilibrium p-value, calculated in our control sample; P-val: p-value for the association under the additive model. +, indel, SNP excluded from analyses, mapping with a triallelic SNP (rs34543648); # SNPs with a MAF<0.05 excluded from analyses. Bonferroni threshold for multiple testing correction p=0.05/34 SNPs

Deleted: polimorphism

Table S4. Description of the summary statistics of publicly available GWAS data of eight psychiatric disorders and the corresponding cross-disorder dataset used for gene-based and gene-set analyses.

GWAS	Participants	Reference
**Attention-Deficit Hyperactivity Disorder, ADHD**	19,099 Ca + 34,194 Co	Demontis et al. 2019 [2]
**Antisocial Behavior, ASB**	16,400	Tielbeek et al, 2017 [3]
**Anxiety**	12,655 Ca + 19,255 Co	Meier et al. 2019 [4]
**Autism Spectrum Disorder, ASD**	18,382 Ca + 27,969 Co	Grove et al. 2019 [5]
**Bipolar Disorder, BD**	20,352 Ca + 31,358 Co	Stahl et al. 2019 [6]
**Major Depressive, MDD**	59,851 Ca + 113,154 Co	Wray et al. 2018 [7]
**Obsessive-Compulsive Disorder, OCD**	1,773 Ca + 6,122 Co + 915 trios	Arnold et al. 2018 [8]
**Schizophrenia, SCZ**	32,405 Ca + 42,221 Co + 1,235 trios	Ripke et al. 2014 [9]
**Cross-Disorder meta-analysis**	162,151 Ca + 276,846 Co	Lee et al. 2019 [10]

Ca: Cases; Co: controls.

Table S5. Experimental design of targeted next-generation sequencing: The coding regions of 14-3-3 genes was covered by 57 amplicons.

Request_ID	Target_ID	Gene	Chr:start-end	N Amplicons	Total Bases	Covered Bases	Missed Bases	Coverage
**IAD38961**	503029	*YWHAE*	chr17:1248735-98	1	64	64	0	1
**IAD38961**	503026	*YWHAE*	chr17:1257499-646	2	148	148	0	1
**IAD38961**	503030	*YWHAE*	chr17:1264380-597	3	218	218	0	1
**IAD38961**	503028	*YWHAE*	chr17:1265190-307	2	118	118	0	1
**IAD38961**	503025	*YWHAE*	chr17:1268147-357	2	211	211	0	1
**IAD38961**	503027	*YWHAE*	chr17:1303335-409	1	75	75	0	1
**IAD38961**	484571	*YWHAQ*	chr2:9725409-479	1	71	71	0	1
**IAD38961**	484573	*YWHAQ*	chr2:9727537-643	2	107	107	0	1
**IAD38961**	484570	*YWHAQ*	chr2:9728288-462	2	175	175	0	1
**IAD38961**	484572	*YWHAQ*	chr2:9731515-649	2	135	135	0	1
**IAD38961**	484574	*YWHAQ*	chr2:9770282-586	3	305	305	0	1
**IAD38961**	503001	*SFN*	chr1:27189698-190455	6	758	758	0	1
**IAD38961**	484575	*YWHAH*	chr22:32340714-811	1	98	72	26	0.735
**IAD38961**	484576	*YWHAH*	chr22:32352120-2784	5	665	665	0	1
**IAD38961**	482745	*YWHAB*	chr20:43530169-0479	3	311	311	0	1
**IAD38961**	482743	*YWHAB*	chr20:43532628-762	2	135	135	0	1
**IAD38961**	482744	*YWHAB*	chr20:43533603-777	2	175	175	0	1
**IAD38961**	482742	*YWHAB*	chr20:43534636-742	1	107	107	0	1
**IAD38961**	482741	*YWHAB*	chr20:43535017-084	1	68	68	0	1
**IAD38961**	482387	*YWHAG*	chr7:75958888-9555	5	668	668	0	1
**IAD38961**	482388	*YWHAG*	chr7:75988033-130	1	98	98	0	1
**IAD38961**	482475	*YWHAZ*	chr8:101932915-85	1	71	71	0	1
**IAD38961**	482472	*YWHAZ*	chr8:101936177-283	2	107	107	0	1
**IAD38961**	482473	*YWHAZ*	chr8:101936357-531	1	175	53	122	0.303
**IAD38961**	482471	*YWHAZ*	chr8:101937138-272	2	135	135	0	1
**IAD38961**	482474	*YWHAZ*	chr8:101960818-1122	3	305	305	0	1

For each coding exon the number of amplicons used to cover the exon length is reported. The overall coverage was 96.3% across the 14-3-3 gene family.

Table S6. Gene-set association results of the 14-3-3 family set of genes on eight different psychiatric phenotypes and in the cross-disorder meta-analysis dataset.

	ADHD	ASB	Anxiety	ASD	BP	MDD	OCD	**SCZ**	Cross-disorder
p value	0.251	0.476	0.270	0.835	0.114	0.757	0.090	**0.018**	0.112

p-values were calculated using MAGMA (v1.06) software. Nominal association is highlighted in bold. ADHD: attention-deficit hyperactivity disorder; ASB: antisocial behaviour; ASD: autism spectrum disorder; BP: bipolar disorder; MDD: major depression disorder; OCD: obsessive-compulsive disorder; SCZ: schizophrenia.

Table S7. Rare variants identified in the seven 14-3-3 family genes in 288 European ASD patients.

Individual	Sex	Comorbidity	Family type	Origin	Gene	Allelic change	Chr:position[a]	gene region	Protein	dbSNP	SIFT/ Provean[b]	PolyPhen2 [c]	MAF (ExAC)[d]
MT_37.3	M	-	S	mother	*SFN*	A/T	1:27190196	exon	p.T165S	rs77755255	**Damaging / Deleterious**	**Probably damaging**	2.47E-04
				mother	*SFN*	GGA/-	1:27189925-27189927	exon	p.E76del	-	**- / Deleterious**	-	1.65E-05
				mother	*SFN*	C/T	1:27190149	exon	p.S149L	rs78707984	Tolerated / Neutral	benign	2.48E-04
MT_11.3	M	LD	S	unknown	*SFN*	C/T	1:27189940	exon	p.P79P	-	Tolerated / Neutral	-	-
MT_159.3	M	-	S	unknown	*YWHAE*	G/A	17:1248772	exon; ncRNA	p.A246V	-	Tolerated / Neutral	benign	-
10-09471	M	-		unknown	*YWHAE*	G/A	17:1303445	5'UTR, ncRNA	-	rs139532375	-	-	3.56E-03
SJD_64.3	M	-	S	mother	*YWHAB*	A/C	20:43530403	exon	p.K77Q	rs142757633	Damaging / Neutral	benign	1.82E-03
SJD_18.3	M	-		father	*YWHAB*	G/A	20:43532595	intronic	-	rs199806929	-	-	2.61E-03
MT_160.3	M	Epilepsy	Mx	mother	*YWHAZ*	ins-T	8:101936203–204	exon	p.L220F*fs**18	-	-	-	-

[a]GRCh37/hg19 assembly; [b]SIFT/Provean (Sorting Intolerant from Tolerant, http://sift.jcvi.org/); [c]PolyPhen2 (http://genetics.bwh.harvard.edu/pph2); [d]MAF (Minor Allele Frequency) of the variant in the ExAC Browser (http://exac.broadinstitute.org);). M: male; Mx, multiplex; LD: Language Delay; ncRNA: non-coding RNA; S, singleton; 5'UTR: 5' untranslated region.

SUPPLEMENTARY FIGURES

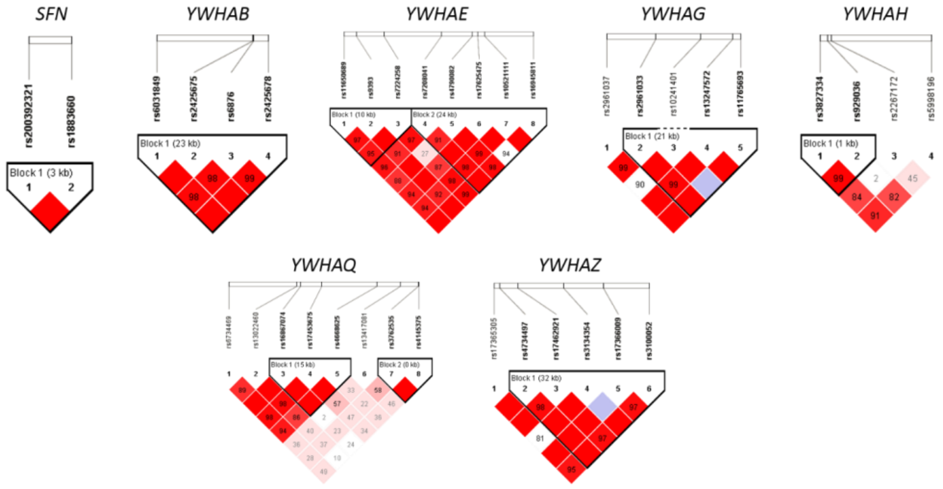

Figure S1. Linkage disequilibrium values among the 37 tagSNPs analyzed in this study, calculated from the whole sample (1441 individuals with European ancestry) with the Haploview software. D' values between all the possible SNP pairs are shown.

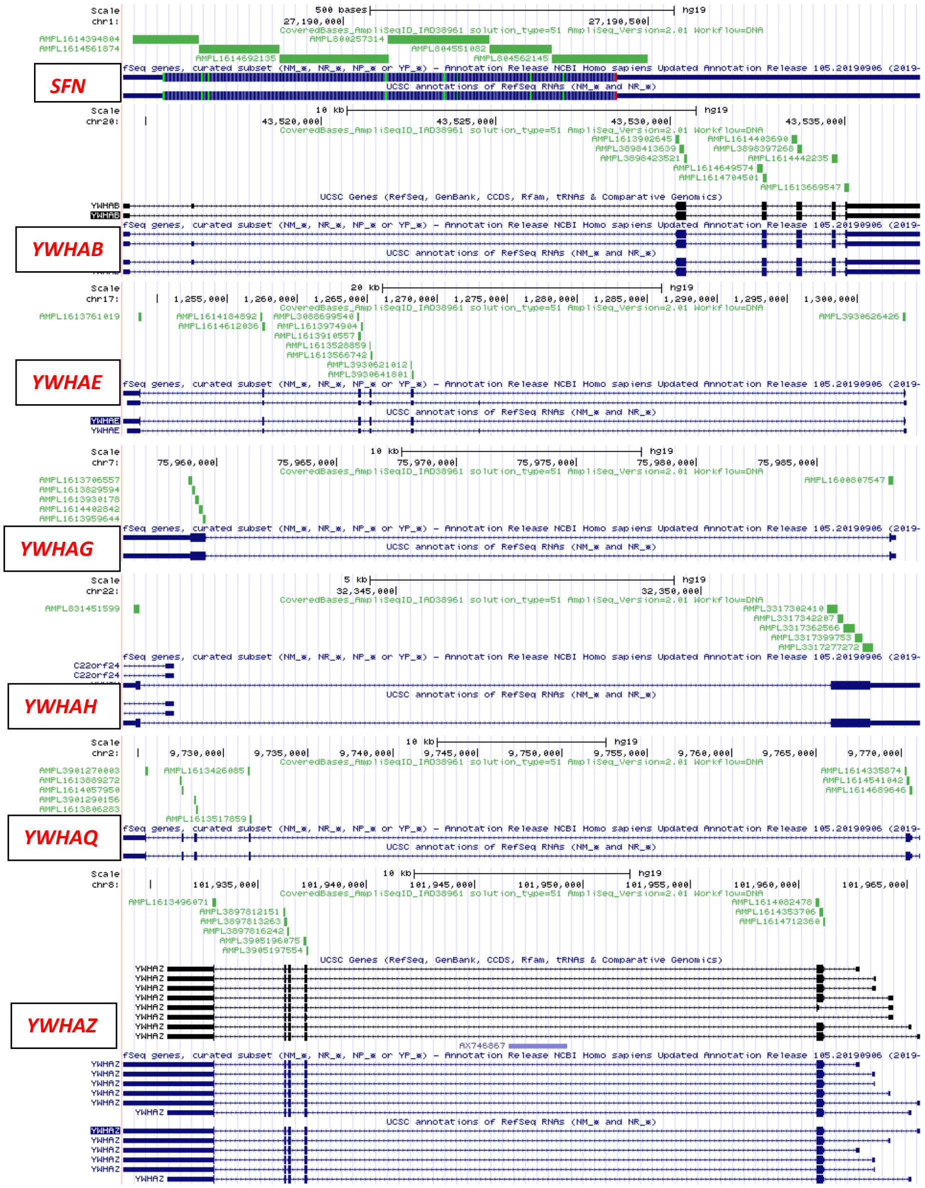

Figure S2. The 57 amplicons used in the mutational screening are depicted in green and cover the coding regions of the 14-3-3 genes. For each of the seven genes we show the amplicon ID name, the number of overlapping amplicons per exon and the genomic region.

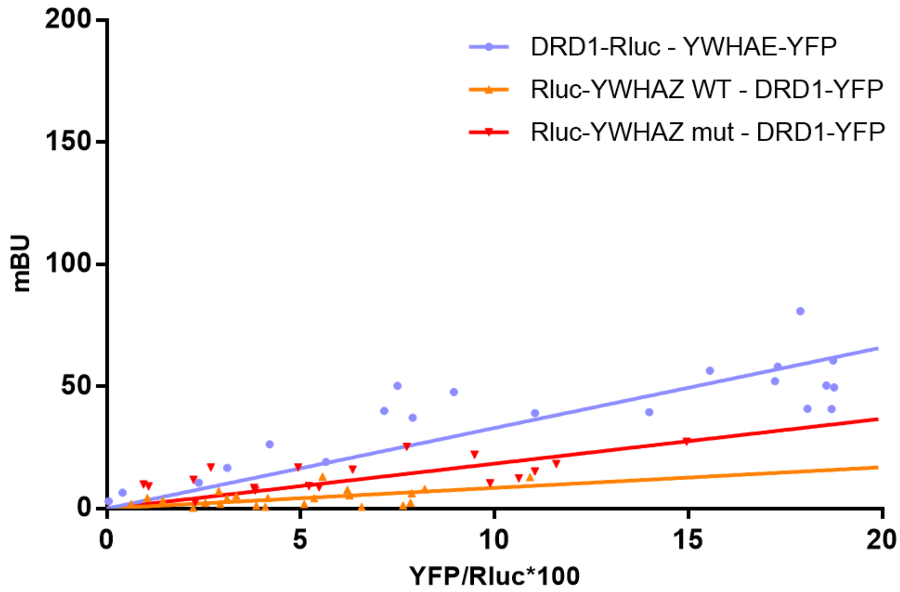

Figure S3. Negative controls in the BRET experiments of the interaction of YWHAZ WT or YWHAZ mutant (mut) with YWHAE using D(1A) dopamine receptor as a donor (DRD1-Rluc) or an acceptor (DRD1-YFP) and adjusted to a linear regression. mBU: BRET ratio expressed in milli-BRET units. The relative amount of BRET is given as a function of YFP/Rluc*100. YFP corresponds to the fluorescence signal due to the increasing amount of donor and Rluc corresponds to the stable luminescence signal measured at 10 minutes. BRET values shown correspond to 3-4 independent experiments.

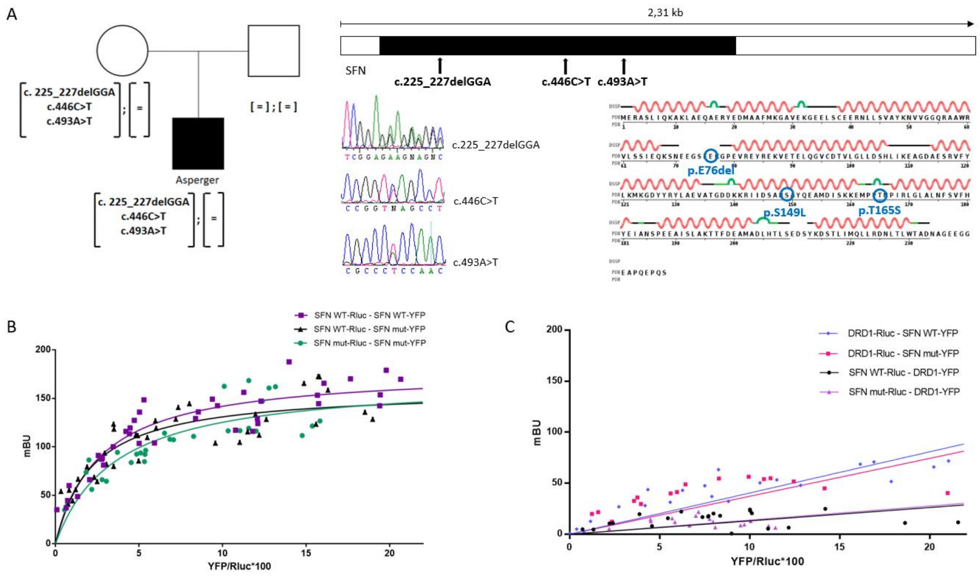

Figure S4. Characterization of three rare inherited *SFN* variants identified in an ASD patient A) Pedigree of the family carrying the mutations. [=], wild-type allele and location of the mutations in the *SFN* gene, Sanger sequences corresponding to the three mutations identified in the patient, and SFN protein sequence together with the secondary structure showing the location of the amino acid changes in the mutant protein. In blue, mutations found in the patient. Protein secondary structure obtained from the Research Collaboratory for Structural Bioinformatics Protein Data Bank (RCSB PDB, http://www.rcsb.org/). Protein secondary structure legend: green curve, turn; green line, bend; red, alpha helix, empty or black line, no secondary structure assigned. B) BRET assay to determine the ability to form homodimers of wild-type SFN (SFN WT) and mutant SFN (SFN mut) co-transfected in HEK293T cells. Values shown correspond to 3-7 different experiments performed. $BRET_{50}$ (SFN mut-Rluc - SFN mut-YFP: 3.57±0.89; SFN WT-Rluc - SFN mut-YFP: 2.18±0.42; SFN WT-Rluc - SFN WT-YFP: 2.66±0.43) and BRETmax (SFN mut-Rluc - SFN mut-YFP: 170.1±13.83; SFN WT-Rluc - SFN mut-YFP: 159.3±8.35; SFN WT-Rluc - SFN WT-YFP: 179.4±7.78). C) Negative controls of SFN WT and mutant (mut) interaction using D(1A) dopamine receptor as a donor (DRD1-Rluc) or an acceptor (DRD1-YFP) and adjusted to a linear regression. Values shown correspond to 2-6 different experiments performed. mBU: BRET ratio expressed in milli-BRET units. The relative amount of BRET is given as a function of YFP/Rluc*100. YFP correspond to the fluorescence signal due to the increasing amount of donor and Rluc correspond to the stable luminescence signal measured at 10 minutes.

REFERENCES

1. Ghorbani, S.; Fossbakk, A.; Jorge-Finnigan, A.; Flydal, M.I.; Haavik, J.; Kleppe, R. Regulation of tyrosine hydroxylase is preserved across different homo- and heterodimeric 14-3-3 proteins. *Amino Acids* **2016**, *48*, 1221–1229, doi:10.1007/s00726-015-2157-0.

2. Demontis, D.; Walters, R.K.; Martin, J.; Mattheisen, M.; Als, T.D.; Agerbo, E.; Baldursson, G.; Belliveau, R.; Bybjerg-Grauholm, J.; Bækvad-Hansen, M.; et al. Discovery of the first genome-wide significant risk loci for attention deficit/hyperactivity disorder. *Nat. Genet.* **2019**, *51*, 63–75, doi:10.1038/s41588-018-0269-7.

3. Tielbeek, J.J.; Johansson, A.; Polderman, T.J.C.; Rautiainen, M.R.; Jansen, P.; Taylor, M.; Tong, X.; Lu, Q.; Burt, A.S.; Tiemeier, H.; et al. Genome-wide association studies of a broad spectrum of antisocial behavior. *JAMA Psychiatry* **2017**, *74*, 1242–1250, doi:10.1001/jamapsychiatry.2017.3069.

4. Meier, S.M.; Trontti, K.; Purves, K.L.; Als, T.D.; Grove, J.; Laine, M.; Pedersen, M.G.; Bybjerg-Grauholm, J.; Bækved-Hansen, M.; Sokolowska, E.; et al. Genetic Variants Associated with Anxiety and Stress-Related Disorders: A Genome-Wide Association Study and Mouse-Model Study. *JAMA Psychiatry* **2019**, *76*, 924–932, doi:10.1001/jamapsychiatry.2019.1119.

5. Grove, J.; Ripke, S.; Als, T.D.; Mattheisen, M.; Walters, R.K.; Won, H.; Pallesen, J.; Agerbo, E.; Andreassen, O.A.; Anney, R.; et al. Identification of common genetic risk variants for autism spectrum disorder. *Nat. Genet.* **2019**, *51*, 431–444, doi:10.1038/s41588-019-0344-8.

6. Stahl, E.A.; Breen, G.; Forstner, A.J.; McQuillin, A.; Ripke, S.; Trubetskoy, V.; Mattheisen, M.; Wang, Y.; Coleman, J.R.I.; Gaspar, H.A.; et al. Genome-wide association study identifies 30 loci associated with bipolar disorder. *Nat. Genet.* **2019**, *51*, 793–803, doi:10.1038/s41588-019-0397-8.

7. Wray, N.R.; Ripke, S.; Mattheisen, M.; Trzaskowski, M.; Byrne, E.M.; Abdellaoui, A.; Adams, M.J.; Agerbo, E.; Air, T.M.; Andlauer, T.M.F.; et al. Genome-wide association analyses identify 44 risk variants and refine the genetic architecture of major depression. *Nat. Genet.* **2018**, *50*, 668–681, doi:10.1038/s41588-018-0090-3.

8. Arnold, P.D.; Askland, K.D.; Barlassina, C.; Bellodi, L.; Bienvenu, O.J.; Black, D.; Bloch, M.; Brentani, H.; Burton, C.L.; Camarena, B.; et al. Revealing the complex genetic architecture of obsessive-compulsive disorder using meta-analysis. *Mol. Psychiatry* **2018**, *23*, 1181–1188, doi:10.1038/mp.2017.154.

9. Ripke, S.; Neale, B.M.; Corvin, A.; Walters, J.T.R.; Farh, K.H.; Holmans, P.A.; Lee, P.; Bulik-Sullivan, B.; Collier, D.A.; Huang, H.; et al. Biological insights from 108 schizophrenia-associated genetic loci. *Nature* **2014**, *511*, 421–427, doi:10.1038/nature13595.

10. Lee, P.H.; Anttila, V.; Erneri; Won, H.; Feng, Y.-C.A.; Rosenthal, J.; Zhu, Z.; Tucker-Drob, E.M.; Nivard, M.G.; Grotzinger, A.D.; et al. Genomic Relationships, Novel Loci, and Pleiotropic Mechanisms across Eight Psychiatric Disorders. *Cell* **2019**, *179*, 1469-1482.e11, doi:10.1016/j.cell.2019.11.020.
